# Determinants of coexisting forms of undernutrition among under‐five children: Evidence from the Bangladesh demographic and health surveys

**DOI:** 10.1002/fsn3.3484

**Published:** 2023-06-09

**Authors:** Imran Hossain Sumon, Moyazzem Hossain, Sifat Ar Salan, Mohammad Alamgir Kabir, Ajit Kumar Majumder

**Affiliations:** ^1^ Department of Statistics Jahangirnagar University Savar, Dhaka Bangladesh

**Keywords:** BDHS‐2017‐2018, logistic regression, pediatric undernutrition, under‐five children

## Abstract

In many underdeveloped and developing countries, epidemiological and nutritional transitions are leading to an increase in malnutrition, resulting in pediatric diseases and eventually deaths. Therefore, this study intents to determine the important factors of the presence of coexisting forms of malnutrition (CFM), i.e., pediatric undernutrition. This study used the latest Bangladesh Demographic and Health Survey (BDHS)‐2017/18 dataset consisting of 7127 under‐five children. The logistic regression model has been utilized to gain explicit and in‐depth knowledge of the relationship between the presence of pediatric undernutrition with socioeconomic and demographic factors. Findings revealed that about 31%, 22%, and 8% suffered from stunted, underweight, and wasted, respectively. The prevalence of stunted, underweighted, wasted, and CFM among children in the Sylhet division is higher than in any other region. A child of a secondary‐level completed mother is 27.6% (OR: 0.724, 95% CI: 0.58–0.90) less likely to suffer from undernutrition than a child of an uneducated mother. The rate of undernutrition of children was less among children of highly educated parents. Age, birth order of the child, twin status, mother's age, body mass index (BMI), working status, parental educational qualification, cooking fuel, toilet facility, region, residence, and wealth index are important for determining the nutritional status of a child. The authors believe that the study findings will be helpful to the policymakers to take proper actions for achieving the sustainable development goal (SDGs) by reducing pediatric undernutrition in Bangladesh by 2030.

## Graphical abstract

This study used the latest Bangladesh Demographic and Health Survey (BDHS)‐2017/18 dataset consisting of 7127 under‐five children. The prevalence of stunted, underweight, and wasted children in the Sylhet division is higher than in any other region. The rate of malnutrition in children of highly educated parents was low.

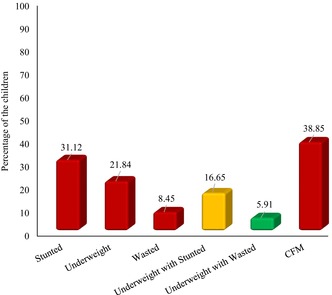

## 1 BACKGROUND

Malnutrition is a term used to describe both overnutrition and undernutrition of individuals that are the barriers to good health (Katsilambros et al., 2011). It is a common problem among the children of developing countries resulting in both long‐ and short‐term consequences such as cognitive impairment (i.e., stunted), underweight, wasted, and eventually death (Katsilambros et al., 2011). Undernutrition (i.e., deficiency of nutrition) can result in being underweight, wasted, and an impediment to growth. However, according to the information from a previous study, developing countries have experienced overnutrition in the mold of obesity like undernutrition (Wells et al., 2020). It is difficult to differentiate between undernutrition and overnutrition by using malnutrition instead of undernutrition. Generally, the term malnutrition is used in most clinical studies to refer to undernutrition (Hickson & Smith, 2018; Ngaruiya et al., 2017). Undernutrition is still a serious problem, especially for young children under the age of five (Popkin et al., 2012). In 2018, approximately 3.1 million children aged less than 5 years die in a single year due to malnutrition (UNICEF., 2018). In 2021, of the five million child deaths, about 2.25 million (i.e., 45%) are due to malnutrition (WHO, 2021).

The prevalence of undernutrition among under‐five children is influenced by several socioeconomic, demographic, environmental, and other factors. Previous studies highlighted that the height of the mother, household food insecurity, residence, region, age of the mother, and socioeconomic status of a family had a significant effect on malnutritional status (Félix‐Beltrán et al., 2021; Rahman & Hossain, 2022). Previous studies conducted on demographic and health survey data have revealed that several sociodemographic and socioeconomic factors are the influential determinants of child malnutrition (Abdulla, El‐Raouf, et al., 2022; Hossain et al., 2023; Hossain et al., 2021; Panda et al., 2020; Perez‐Escamilla et al., 2018; Rahman et al., 2021; Rahman et al., 2020; Sazedur et al., 2020). The growth in overweight and obesity in Bangladesh is a result of the country's population shifting from a traditional diet of rice, vegetables, and lentils to one that is more Westernized and includes processed foods, sugary beverages, and snacks (Popkin et al., 2012). However, there are some adverse impacts of malnutrition on child health status. In order to enrich the adverse effects on child health and fatality, colossal attention must be paid to the identification of covariates involved with malnutrition. Despite widespread worldwide accentuation on the nutritional status of children, the progression of mortality rates is declining very slowly. An earlier study had shown that exclusive breastfeeding and diarrhea were closely linked to the problem of wasting in rural areas, whereas only exclusive breastfeeding was closely associated with wasting in urban slum children (Murarkar et al., 2020). A study highlighted that insufficient exclusive breastfeeding up to 6 months of age may increase the risk of child morbidity (Abdulla, Hossain, et al., 2022). Furthermore, the presence of more than one type of nutritional disorder, i.e., Coexisting Forms of Malnutrition (CFM), is more at risk of dying than children who just have one kind of malnutrition (Garenne et al., 2019; Khaliq, Wraith, Nambiar, & Miller, 2022). Several studies pointed out that age, sex, birth order, and birth size of children, diet, health and illness status, parent's education, maternal age and occupation, family size, socioeconomic status, water source, and toilet facility are associated with CFM (Farah et al., 2021; Fernald & Neufeld, 2007; Fongar et al., 2019; Islam & Biswas, 2020; Khaliq et al., 2021; Rachmi et al., 2016; Roba et al., 2021; Saaka & Galaa, 2016; Varghese & Stein, 2019; Zhang et al., 2016).

Most of the previous studies used different statistical models to identify important determinants related to any one of the three indicators of malnutrition. Other remaining studies focused to identify factors linked to the double burden of malnutrition. Earlier research also explored the prevalence, patterns, and factors that cause the coexisting forms of malnutrition in neonates, infants, and children (Khaliq et al., 2021; Khaliq, Wraith, Miller, & Nambiar, 2022; Khaliq, Wraith, Nambiar, & Miller, 2022). In the context of Bangladesh, there is a lack of evidence focusing on the determinants of the presence of CFM considering the most recent countrywide survey BDHS‐2017/18 data. Therefore, to fill up this backdrop, in this study, any one of the three indicators (stunted, underweight, and wasted) or the CFM was considered as the target variable. The main objective of the present study is to identify the important determinants associated with a dichotomous variable created by the combination of the three indicators of pediatric undernutrition that will be beneficial to the policymakers for taking proper plans and actions in order to achieve the Sustainable Development Goals (SDGs) in Bangladesh by 2030.

## 2 METHODS

### 2.1 Data source

The study is based on the secondary dataset extracted from the 8th National Demographic and Health Survey (BDHS)‐2017/18 in Bangladesh. In the first phase of the two‐stage stratified sample of households used in the survey, 425 rural enumeration units and 250 urban enumeration units were selected by the sampling technique of probability proportional size. For the purpose of family selection in the second phase, a complete list of all the households in the selected enumeration unit was prepared in the first phase. In the second phase, 30 households were selected from each enumeration unit using a systematic sampling method. This survey was carried out with 8772 children in the remaining 672 clusters, excluding one urban cluster in Dhaka, one rural cluster in Rangpur, and one rural cluster in Rajshahi region due to natural disasters. There were 7127 children under the age of five in the final sample due to the elimination of missing observations. In the study, appropriate weight has been used prior to the analysis to confirm the representative sample of the country. The BDHS‐2017/18 report discusses, in detail, about the sampling procedures, and guidelines for the use of weight representing the country (National Institute of Population Research and Training (NIPORT) & ICF, 2020).

### 2.2 Target variable

Based on children's height for age, stunting (i.e., low height‐for‐age Z(HAZ) score) is an assessment of chronic malnutrition. Based on weight for height, wasting (i.e., low weight‐for‐height Z (WHZ) score) is a measure of excess nutrient deficiency. Based on weight for age, underweight (i.e., low weight‐for‐age Z(WAZ) score) is a composite measure of both overweight and chronic conditions. These were enumerated by Z‐score (i.e., standardized score) based on WHO 2006 child growth standards (Rutstein & Rojas, 2006). The Z‐score for the $i^{\mathrm{th}}$ child ($Z_{i}$) is defined as $Z_{i}=\frac{X_{i}-\mu }{\sigma }$, where $X_{i}$ is underweight, stunting, and wasting of the $i^{\mathrm{th}}$ child and μ and σ indicate the median and SD (i.e., standard deviation), respectively (Asmare & Agmas, 2022; Hosen et al., 2023). After the $Z_{i}$ for each child is calculated, the status of stunted, wasted, and underweight was recoded into dichotomies as follows:

$$\text{Stunted}=\left\{\begin{array}{c} 0="\mathrm{No}"\;\text{if}\;\mathrm{HAZ}\geq -2\\ 1="\mathrm{Yes}"\;\text{if}\;\mathrm{HAZ}<-2 \end{array}\right.$$

$$\text{Wasted}=\left\{\begin{array}{c} 0="\mathrm{No}"\;\text{if}\;\mathrm{WHZ}\geq -2\\ 1="\mathrm{Yes}"\;\text{if}\;\mathrm{WHZ}<-2 \end{array}\right.$$

$$\text{Underweight}=\left\{\begin{array}{c} 0="\mathrm{No}"\;\text{if}\;\mathrm{WAZ}\geq -2\\ 1="\mathrm{Yes}"\;\text{if}\;\mathrm{WAZ}<-2 \end{array}\right.$$

Finally, the CFM can be obtained as follows.

$$\mathrm{CFM}=\left\{\begin{array}{c} 0="\mathrm{No}"\;\text{if Stunted}+\text{Wasted}+\text{Underweight}=0\\ 1="\mathrm{Yes}"\;\;\text{if Stunted}+\text{Wasted}+\text{Underweight}>0 \end{array}\right.$$

However, researchers may consider any two from stunted, wasted, and underweight to define the CFM for their study purpose.

### 2.3 Covariates

In this study, socioeconomic and demographic features such as sex of children (female and male), age of children (below 6, 6–23.9, and 24+ months), birth order (first, second–third, at least fourth), twin status (single birth, first of multiple births, and second of multiple births), maternal current age in years (15–19, 20–24, 25–34, 35–49 years), maternal age at marriage (15–19, 20–24, 25–34, 35–49 years), maternal age at first birth (15–19, 20–24, 25–34, 35–49 years), maternal body mass index (underweight, normal, overweight, obesity), mothers education level (no education, primary, secondary, and higher than secondary), working condition of mother (yes, no), father's education level (no education, primary, secondary, and higher than secondary), household wealth exponent (poorer, poorest, middle, richer, richest), receiving vitamin A (yes, no), toilet facility (improved toilet, unimproved toilet), water supply (improved toilet, unimproved toilet), cooking fuel (gas/electricity, wood, others), residence (rural, urban), and region (Dhaka, Barisal, Chattogram, Khulna, Rajshahi, Rangpur, Mymensingh, and Sylhet) are regarded as relevant covariates. Several dimensions such as appearance in the dataset BDHS 2017–2018, relevant literature, and self‐consideration influence the selection of the covariates used in this study.

### 2.4 Statistical methods

This study uses the $\chi^{2}$ test as a tool for bivariate analysis to determine influential socioeconomic and demographic covariates (e.g., independent variables for logistic regression) of multiple burdens of malnutrition. Logistic regression analysis is performed to narrate the influence of sociodemographic features on multiple burdens of malnutrition in Bangladesh. Joseph Berkson developed and promoted logistics regression as a statistical model (Cramer, 2002). The form of the logistic regression in matrix notation is as follows:

$$\log\left(\frac{p}{1-p}\right)=\mathrm{X\beta}+\varepsilon ,$$

$$\beta =\left[\begin{array}{c} \beta_{1}\\ \beta_{2}\\ \begin{array}{c} \vdots \\ \beta_{k} \end{array} \end{array}\right];X=\left[\begin{array}{ccc} 1 & x_{11} & \cdots \;x_{k1}\\ 1 & x_{12} & \begin{array}{cc} \cdots & x_{k2} \end{array}\\ \begin{array}{c} \vdots \\ 1 \end{array} & \begin{array}{c} \vdots \\ x_{1n} \end{array} & \begin{array}{cc} \begin{array}{c} \ddots \\ \cdots \end{array} & \begin{array}{c} \vdots \\ x_{\mathrm{kn}} \end{array} \end{array} \end{array}\right]$$

where *X* represents the matrix of covariates, *p* is the probability of occurring an event, and *β* is the vector of regression coefficients.

## 3 RESULTS

**FIGURE 1:**
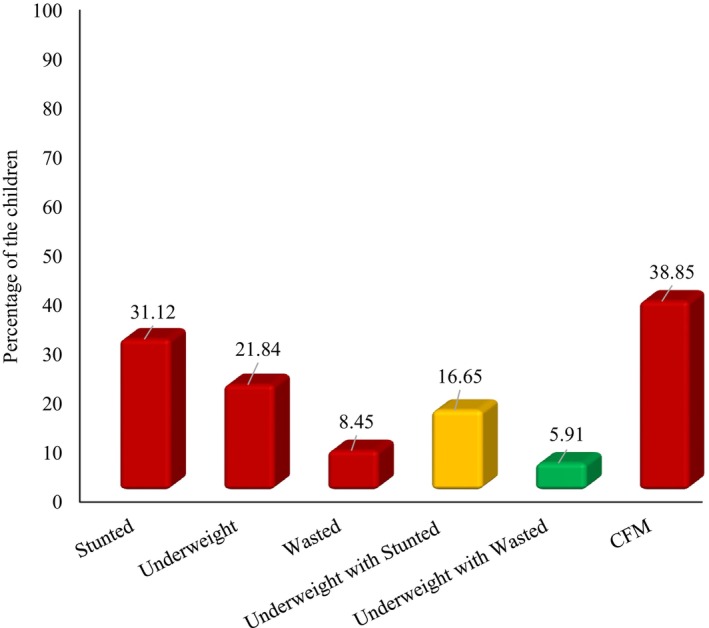
Prevalence of possible combination of stunted, underweight, and wasted children.

The goal of this study was to determine the prevalence of coexisting forms of malnutrition among Bangladeshi children under the age of five. The prevalence of stunted, underweight, and wasted children and their composition are presented in Figure 1.

In Bangladesh, 38.9% of children aged below 5 years have CFM (Figure 1) of which 16.7% have coexistence of underweight with stunting, 5.9% has coexistence of underweight with wasting, and 2.8% has underweight with both wasting and stunting. Figure 1 also depicts that about 31%, 22%, and 8% suffered from stunted, underweight, and wasted, respectively. Moreover, about 17% of children suffered from the coexistence of underweight with stunting, a small proportion of children suffered from the coexistence of stunting with wasting (2.82%) and approximately 6% of children were faced concurrent underweight with wasting, and more than 38% of Bangladeshi under‐five children suffered from coexistence of underweight with both wasting and stunting (Figure 1).

To determine the significant characteristics of malnutritional status of children, Pearson's Chi‐square tests were performed, and the results are presented in Table 1. Findings revealed that stunted, underweight, and CFM have a significant relationship with the age of the child. Children aged more than 23.9 months suffered more from underweighted but no other forms of malnutrition compared to other age groups. Stunted, underweight, and malnutritional status have a significant relationship with the birth order of the child. A child of higher birth order is more likely to suffer from malnutrition (Table 1).

**TABLE 1:** Percentage distribution of selected covariates with different forms of malnutrition (*n* = 7127).

Characteristics	Form of child malnutrition, (%)			
	Stunted (*n*1 = 2218)	Underweight (*n*2 = 1557)	Wasted (*n*3 = 602)	CFM (*n*4 = 2769)
Sex of child				
Male	31.03	21.54	9.14	38.88
Female	31.24	22.18	7.68	38.82
*p*‐value	.848	.510	.027	.958
Age of child in month				
Below 6	11.48	2.57	2.65	14.35
6–23.9	33.82	22.67	10.16	42.13
Above 23.9	33.37	26.35	8.97	41.48
*p*‐value	<.001	<.001	<.001	<.001
Birth order				
1	29.03	20.16	8.38	36.39
2–3	30.13	21.16	8.65	38.43
At least 4	41.17	29.47	7.84	47.79
*p*‐value	<.001	<.001	.724	<.001
Twin				
Single birth	30.79	21.54	8.40	38.49
First of multiple births	48.00	38.00	9.80	54.90
Second of multiple births	57.14	44.64	14.29	69.09
*p*‐value	<.001	<.001	.272	<.001
Age of mother				
15–19	30.61	21.27	8.91	38.56
20–24	30.05	20.54	8.04	36.77
25–34	31.42	23.44	8.86	39.22
35–49	33.44	27.76	9.20	42.81
*p*‐value	.372	<.001	.629	.032
Age of mother at marriage				
15–19	32.17	23.18	8.67	39.74
20–24	20.56	16.94	8.55	28.45
25–34	16.95	11.86	5.08	22.88
35–49	0.00	0.00	0.00	0.00
*p*‐value	<.001	<.001	.573	<.001
Age of mother at first birth				
15–19	33.450	24.44	9.20	41.30
20–24	26.63	18.65	7.54	33.33
25–34	19.79	14.97	5.84	27.41
35–49	0.00	13.33	6.67	13.33
*p*‐value	<.001	<.001	.026	<.001
Mother's body mass				
Underweight (Below 18.50)	40.82	31.44	13.77	50.75
Normal (18.50–24.99)	32.15	22.22	8.01	39.92
Overweight (25.00–29.99)	23.53	16.46	7.14	30.40
Obesity (Above 30.00)	22.19	12.10	4.03	26.51
*p*‐value	<.001	<.001	<.001	<.001
Mother's educational status				
No education	44.42	36.05	11.35	53.59
Primary	39.05	26.47	8.97	46.27
Secondary	29.35	20.38	8.48	37.43
Higher	15.28	10.94	5.94	22.17
*p*‐value	<.001	<.001	.002	<.001
Mother's working status				
No	28.74	20.56	8.43	36.89
Yes	34.51	23.63	8.47	41.59
*p*‐value	<.001	.002	.943	<.001
Father's educational status				
No education	43.14	30.62	9.41	50.82
Primary	35.92	24.29	8.51	43.13
Secondary	27.58	19.77	9.01	35.98
Higher	16.93	12.64	6.32	24.43
*p*‐value	<.001	<.001	.027	<.001
Wealth index combined				
Poorest	40.49	28.65	9.68	48.50
Poorer	37.23	25.13	8.62	44.25
Middle	30.17	19.69	7.57	36.26
Richer	26.64	20.66	8.72	36.17
Richest	17.60	12.79	7.33	25.65
*p*‐value	<.001	<.001	.154	<.001
Receiving vitamin A				
No	30.58	21.84	8.69	38.48
Yes	33.68	21.86	7.32	40.63
*p*‐value	.032	.984	.114	.158
Toilet facility				
Improved	28.06	20.51	8.29	35.40
Unimproved	34.88	25.13	9.02	42.74
*p*‐value	<.001	<.001	.266	<.001
Water facility				
Improved	27.81	18.15	7.10	33.93
Unimproved	31.49	23.17	8.84	39.28
*p*‐value	.02	<.001	.07	<.001
Cooking Fuel				
Gas/Electricity	22.91	17.47	9.38	31.75
Wood	33.63	23.56	8.32	41.15
Others	27.11	17.47	7.95	34.50
*p*‐value	<.001	<.001	.430	<.001
Place of residence				
Urban	26.07	19.58	9.07	34.46
Rural	32.78	22.57	8.24	40.28
*p*‐value	<.001	.009	.280	<.001
Region				
Barisal	33.25	22.52	8.93	41.19
Chittagong	32.73	21.32	7.76	39.87
Dhaka	25.06	18.50	8.88	33.65
Khulna	25.40	18.60	8.58	32.70
Mymensingh	36.04	26.32	9.57	43.29
Rajshahi	31.95	22.24	7.36	39.71
Rangpur	30.78	20.52	7.17	37.33
Sylhet	43.41	32.33	10.50	52.42
*p*‐value	<.001	<.001	.272	<.001

Stunted, underweight, wasted, and CFM have significant relationships with the mother's age as well as body mass index. The study revealed that 33.44%, 27.76%, 9.20%, and 42.81% of children of mothers aged more than 39 years suffer from stunted, underweight, wasted, and CFM. Children are suffered more from malnutrition whose mother's BMI belongs to underweighted categories. However, children of obese mothers had less prevalence of malnutrition. Different forms of malnutrition such as stunted, underweight, and CFM are significantly associated with the mother's age at marriage as well as the mother's age at first birth. Stunted, underweight, wasted, and either had CFM status are significantly associated with the educational qualification of parents. In the case of uneducated mothers, the proportion of stunted, underweight, wasted, and CFM child is 44.42%, 36.05%, 11.35%, and 53.59%, respectively. About half of the children suffered from CFM whose mother has no formal education. Findings depict that higher educated mother has fewer malnutritional children. Moreover, results exhibited that stunted, underweight, and malnutritional status have significantly been influenced by mothers' working status, toilet facility, cooking fuel, and wealth index of the families. Table 1 demonstrates that stunted, underweight, and CFM status have significantly been influenced by the place of residence. Child malnutrition is higher in rural areas than in urban areas. In urban areas, the proportion of stunted, underweight, wasted, and CFM child is 26.07%, 19.58%, 9.07%, and 34.46%, respectively, whereas in rural areas, the prevalence of stunted, underweight, wasted, and the presence of CFM is 32.78%, 22.57%, 8.24%, 40.28%, respectively (Table 1).

**FIGURE 2:**
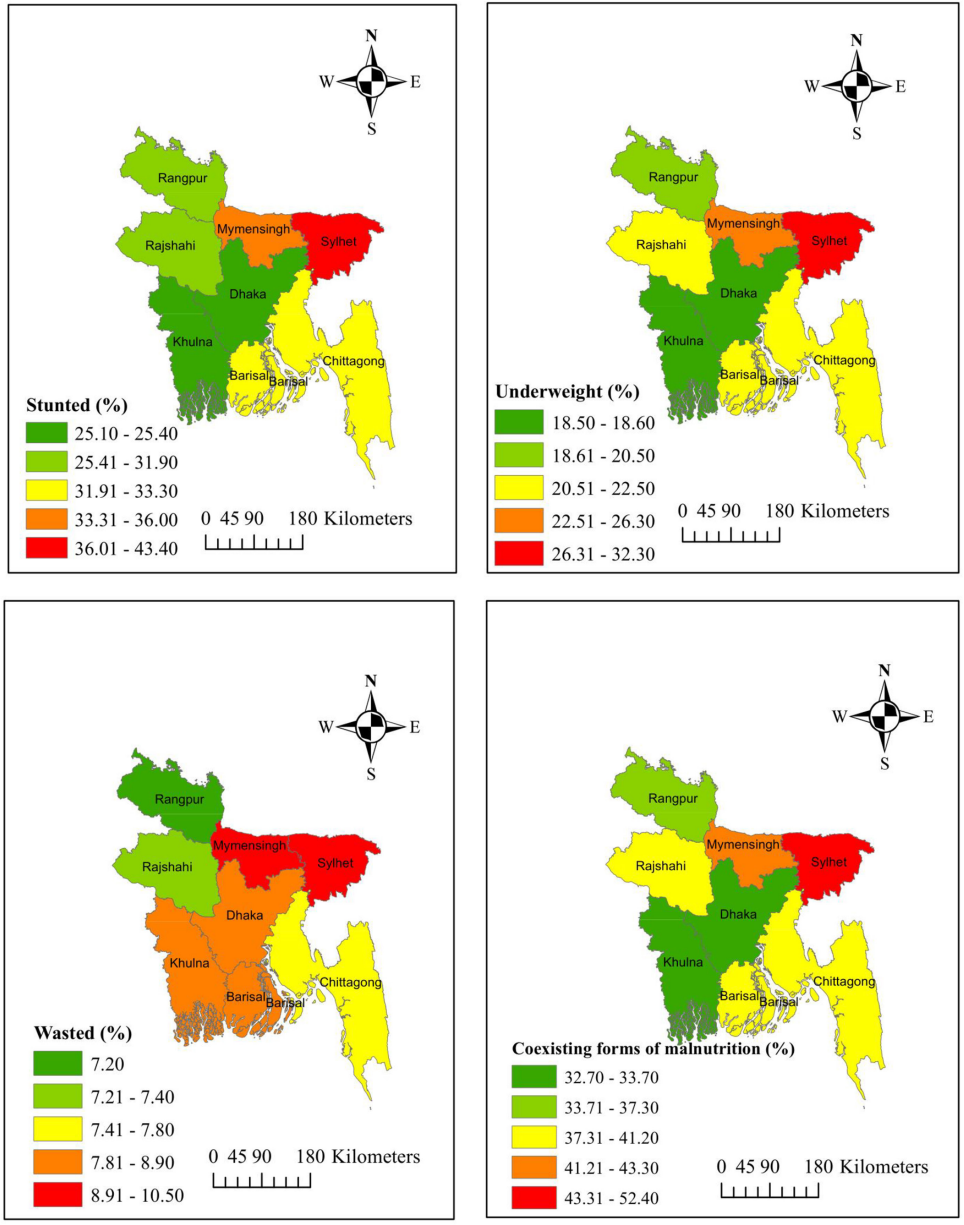
Prevalence of different forms of malnutrition among under‐five children in Bangladesh. Top left: stunted; top right: underweight; bottom left: wasted; bottom right: coexisting forms of malnutrition.

Furthermore, stunted, underweight, wasted, and CFM status have a significant association with toilet type and water facility. Children from households using unimproved toilets and untreated water are more likely to suffer from stunting, underweight, wasting, and CFM than children from households using improved toilets and water. Also, stunted, underweight, and CFM status have a significant relationship with the region. The spatial distributions of different types of malnutrition among children are illustrated in Figure 2. The rate of stunted children is higher than that of both wasted and underweight children. The prevalence of stunted children in Dhaka and Khulna region ranges between 25.10% and 25.40% which is less than in any other region. The rate of stunted children in Sylhet is higher than in any other region which is between 36.01% and 43.40%. The prevalence of underweight children in Dhaka and Khulna region is between 18.50% and 18.60% which is less than in any other region. The rate of underweight children in Sylhet is higher than in any other region which is between 26.31% and 32.30%. No significant association is exhibited between wasted and region. The prevalence of wasted children was more in Mymensingh and Sylhet divisions, whereas the prevalence of wasted children was less observed in Rajshahi and Rangpur divisions. The prevalence of CFM and underweight in different regions has a similarity which is quite different from the prevalence of wasted; however, it is very similar to the prevalence of stunted children. Prevalence of CFM has been reported in 32.70%–33.70% of children in Dhaka and Khulna regions which is lower than in any other region. Prevalence of CFM has been reported in 33.71%–37.30% of children in Rangpur district. The rate of CFM among the children of Barisal, Chittagong, and Rajshahi is less than in the Mymensingh region. Prevalence of CFM has been reported in 43.31%–52.40% of children in the Sylhet region which is higher compared to other regions of Bangladesh (Figure 2 and Table 1).

It is clear that the odds of malnutrition increase with age. Compared to children aged below 6 months, children over the age of 6 months have four folds higher odds of CFM. A child born in multiple births for the first time is 1.58 times more probable to have malnutrition than a child born alone. Moreover, a child born in multiple births for the second time is 1.53 times higher likelihood to have malnourished than a child born alone (Table 2).

**TABLE 2:** Regression coefficients, adjusted odds ratios (OR), and 95% CI of odds ratios (OR) of logistic regression for CFM in Bangladesh.

Characteristics	Coefficients	OR	CI of OR
Age of Child in month			
Below 6 (Ref.)	‐		‐
6–23.9	1.463***	4.320	[3.48, 5.36]
Above 23.9	1.492***	4.445	[3.59, 5.50]
Birth order			
1 (Ref.)	‐		‐
2–3	−0.064	0.938	[0.81, 1.09]
4 or more	0.077	1.080	[0.86, 1.37]
Twin			
Single birth (Ref.)	‐		‐
First multiple births	0.946***	2.575	[1.45, 4.59]
Second multiple births	0.926***	2.526	[1.46, 4.37]
Age of mother			
15–19 (Ref.)			
20–24	−0.035	0.966	[0.81, 1.15]
25–34	0.012	1.013	[0.81, 1.26]
35–49	−0.019	0.982	[0.72, 1.34]
Age of mother at marriage			
15–19 (Ref.)	‐		‐
20–24	−0.057	0.944	[0.74, 1.21]
25–34	0.398	1.489	[0.82, 2.71]
35–49	−18.352	0.000	[0.00, −]
Age of mother at first birth			
15–19 (Ref.)	‐		‐
20–24	−0.053	0.949	[0.82, 1.09]
25–34	−0.157	0.855	[0.60, 1.22]
35–49	−1.120	0.326	[0.07, 1.63]
Mother body mass			
Underweight (Ref.)	‐		‐
Normal	−0.374***	0.688	[0.60, 0.79]
Overweight	−0.658***	0.518	[0.43, 0.62]
Obesity	−0.765***	0.465	[0.35, 0.62]
Mother's educational status			
No education (Re.)	‐		‐
Primary	−0.159*	0.853	[0.69, 1.05]
Secondary	−0.323***	0.724	[0.58, 0.90]
Higher	−0.676***	0.509	[0.39, 0.67]
Mother's working Status			
No (Ref.)	‐		‐
Yes	0.026	1.027	[0.92, 1.14]]
Father's educational status			
No education (Ref.)			
Primary	−0.172**	0.842	[0.72, 0.98]
Secondary	−0.260***	0.771	[0.65, 0.91]
Higher	−0.509***	0.601	[0.48, 0.75]
Wealth index combined			
Poorest (Ref.)	‐		‐
Poorer	−0.093	0.911	[0.79, 1.06]
Middle	−0.209**	0.811	[0.69, 0.96]
Richer	−0.165*	0.848	[0.71, 1.02]
Richest	−0.502***	0.605	[0.48, 0.76]
Toilet facility			
Improved (Ref.)	‐		‐
Unimproved	0.081	1.084	[0.96, 1.22]
Water facility			
Improved (Ref.)			
Unimproved	−0.084	0.920	[0.65, 1.31]
Cooking fuel			
Gas/Electricity (Re.)	‐		‐
Wood	−0.026	0.975	[0.81, 1.17]
Others	−0.289	0.749	[0.50, 1.13]
Place of residence			
Urban (Ref.)	‐		‐
Rural	−0.016	0.984	[0.86, 1.13]
Region			
Barisal (Ref.)	‐		‐
Chittagong	0.114	1.121	[0.89, 1.42]
Dhaka	−0.164	0.849	[0.67, 1.08]
Khulna	−0.241*	0.786	[0.60, 1.03]
Mymensingh	0.038	1.039	[0.80, 1.35]
Rajshahi	0.014	1.014	[0.79,1.30]
Rangpur	−0.167	0.846	[0.66, 1.09]
Sylhet	0.335**	1.397	[1.07, 1.83]
Constant	−0.603	0.547	‐

*p‐value <.10; **p‐value <.05; ***p‐value <.01.

Abbreviation: Ref., Reference category.

Table 2 exhibits that parental characteristics such as the mother's body mass index, the educational qualification of the mother, and the father's educational qualifications are the most important determinant of nutritional status. A child of a normal mother is 31.2% (OR: 0.688, 95% CI: 0.60–0.79) less likely to be malnourished than a child of an underweighted mother. A child of a mother who completed higher education than the secondary level is 49.1% less likely to suffer from malnutrition than a child of an uneducated mother. A child of a father who completed higher education than the secondary level is 39.9% less probable to suffer from malnutrition than a child of an uneducated father. It is apparent that the association between the malnutritional and family wealth index is positive. That is, a child from a family with an improved wealth index is less likely to suffer from malnutrition. A child from a family with the richest wealth index is 39.5% less likely to be malnourished than a child from a family with a poor wealth index. A child who lived in the Sylhet division is more likely to suffer from malnutrition which is 0.397 times more than a child who lived in the Barisal division. A child in the Khulna division has about 21.4% less chance to suffer from malnutrition than a child who lived in the Barisal division (Table 2).

## 4 DISCUSSION

This study identifies the important socioeconomic and sociodemographic determinants of child malnutrition based on BDHS‐2017/18 data that will help the policymakers in Bangladesh. In Bangladesh, the rate of stunted children is considered to be higher than the rate of underweight and wasted problems due to the high incidence of childhood infections, and maternal anxiety during pregnancy. A similar finding is revealed in previous research (Ahmed et al., 2021; Dewey & Begum, 2011; WHO, 2021) and a previous study showed the reverse findings (Querol et al., 2021).

The study findings indicate that the risk of malnutrition increases with children's age. The demand for nutritious food increases with the age of the child but underdeveloped and developing countries are not fully capable to meet the demand for nutritious food which leads to child malnutrition. A previous study in Bangladesh has also corroborated similar relationship (Rahman et al., 2016); however, researchers pointed out that the status of stunting and wasting decreased as the child's age increased (Abdulla et al., 2023; Hossain et al., 2022). While this study shows a significant association between birth order and nutritional status, it does show a negligible difference in nutritional status due to changes in birth order. One of the reasons may be that mothers now place equal importance on all children. Twin children are more likely to suffer from malnutrition than a child born alone because it is very difficult for families to take full care of two children at the same time. A previous study supports these findings (Garanet, 2020). Wasting is an acute form of malnutrition if a mother has to face issues related to twin childcare, then it is expected to have an association between twin children and wasting; however, surprisingly, it is observed an insignificant association between them. Findings revealed that the likelihood of malnutrition is high among children whose parents have no education and children whose parents are highly educated because educated parents have an idea about child malnutrition and take necessary steps. Preceding studies evolved that a child of families with no education is more likely to have malnutrition (Ahmed et al., 2021; Babatunde & Qaim, 2010; Olwedo et al., 2008). A previous study exhibits a similar relationship between the coexistence of underweight with wasting and stunting and the education of mothers (Khaliq, Wraith, Miller, & Nambiar, 2022). This study has shown that the marital age of the mother is significantly associated with child malnutrition which is also corroborated by a survey conducted in rural areas of Nepal (Wells et al., 2022). The prevalence of malnutrition among children in the Sylhet division is higher than in any other region of Bangladesh. Six factors have been identified by experts as the cause of Sylhet's higher child malnutrition incidence. Low household food production rates, inadequate educational attainment, early marriage, a lack of nutrient‐conscious eating practices, a large number of children per family, and low healthcare utilization rates are the reasons (Islam, 2023). Children of working mothers are more likely to have malnourished than the children of nonemployed mothers. One of the logical reasons behind this may be the lack of adequate time for the child due to the busy schedule of working mothers. Similar findings are exhibited in the several preceding studies (Ahmed et al., 2021; Chowdhury et al., 2016). It is a common belief that if a child suffers from different childhood morbidity, the child has the possibility to become a malnourished child. Children of normal, overweight, obese bodies are less likely to be malnourished than children of thin mothers. One of the reasons behind this may be that a malnourished mother gives birth to a malnourished child and a malnourished mother cannot provide adequate breast milk to her child. Previous studies conducted in India (Mishra et al., 2014), Ethiopia (Amare et al., 2019), and Bangladesh (A. Islam & Biswas, 2015; Jesmin et al., 2011; Rahman et al., 2016) have corroborated similar relationship of body mass index of the mother with stunted and underweight.

A child from a family with an improved wealth index is less likely to suffer from malnutrition. A previous study conducted in Pakistan has corroborated a similar relationship of socioeconomic status (i.e., family wealth index) with malnutrition (Khaliq et al., 2021). A study demonstrates that the poorest wealth index was significantly associated with the coexistence of wasting with underweight, coexistence of stunting with underweight, or coexistence of underweight with wasting and stunting (Khaliq, Wraith, Nambiar, & Miller, 2022). This study further showed that no significant change in nutritional status was found for differences in place of residence, cooking fuel, and toilet facility, although Chi‐square tests showed that each covariate was associated with nutritional status. This study further showed that no significant change in nutritional status was found for differences in place of residence, cooking fuel, and toilet facility in regression analysis, although the Chi‐square tests showed that each covariate was associated with nutritional status of children. A plausible reason behind this may be that in Chi‐square test, the authors check the association of each covariate with child malnutrition separately; however, in the regression model, the effects of the covariates are adjusted.

### 4.1 Strength and limitation of the study

The main strengths of this study are that it has been conducted on the most recent representative datasets of the country and originality has been maintained. No previous study has been conducted considering the presence of coexisting forms of malnutrition among under‐five children based on the BDHS‐2017/18 data. This analysis is cross‐sectional, and causal inference is not possible. Moreover, in a future study, different techniques including a multilevel logistic model, and machine learning classifier will be used to mark out the significant determinants of child malnutrition in Bangladesh. Due to the fact that the dependent variable is slightly different in this study compared to the previous study and at the same time higher inclusion, some differences have been observed in the analysis.

## 5 CONCLUSION

Study findings indicate that malnutrition is still a monumental health concern among under‐five children in Bangladesh. Undernutrition in the form of underweight and stunting is more prevalent than wasting in Bangladesh. Among the children of Bangladesh, the prevalence of stunting is higher than two other forms of malnutrition, whereas the prevalence of underweight is more than the form of wasting. Results of this study revealed that the child's age, twin birth status, mother's age at marriage, mother's BMI, educational and working status, father's educational status, and region all are found to be significantly related to childhood malnutrition. Moreover, the association measure exhibits that birth order, mother's age at first birth, toilet type, cooking fuel, place of residence, and family wealth index have a significant relationship with child malnutrition. The authors believe that the study findings will be helpful to the policymakers in order to lessen pediatric undernutrition among children aged below 5 years which will be beneficial to achieve the Sustainable Development Goals (SDGs) by 2030. It would be worthwhile to look into the prevalence, trends, and causes of stunting and wasting, stunting and underweight, wasting and underweight, as well as coexisting forms of undernutrition among under‐five children in Bangladesh in a future study.

## AUTHOR CONTRIBUTIONS

**Imran Hossain Sumon:** Conceptualization (equal); data curation (equal); formal analysis (equal); visualization (equal); writing – original draft (equal); writing – review and editing (equal). **Md. Moyazzem Hossain:** Conceptualization (equal); methodology (equal); supervision (equal); visualization (equal); writing – original draft (equal); writing – review and editing (equal). **Sifat Ar Salan:** Conceptualization (equal); data curation (equal); formal analysis (equal); writing – original draft (equal). **Mohammad Alamgir Kabir:** Conceptualization (equal); supervision (equal); validation (equal); writing – review and editing (equal). **Ajit Kumar Majumder:** Conceptualization (equal); supervision (equal); validation (equal); writing – review and editing (equal).

## FUNDING INFORMATION

All authors ensure that this research received no specific grant from any funding agency in the public, commercial, or not‐for‐profit sectors.

## CONFLICT OF INTEREST STATEMENT

The authors declared no competing interest.

## ETHICAL APPROVAL

The demographic health surveys are available publicly and ethics approvals were completed by institutions that commissioned, funded, and managed the surveys. DHS surveys are approved by Inner City Fund (ICF) International and an in‐country Institutional Review Board (IRB) to ensure that protocols are in compliance with the U.S. Department of Health and Human Services regulations for the protection of human subjects.

## Data Availability

The dataset used in this study will be available from the website of The DHS Program. In order to gain access to the data files, you have to complete the registration. The dataset is available from the following link http://dhsprogram.com/data/available‐datasets.cfm.
